# Do checkpoint inhibitors compromise the cancer patients’ immunity and increase the vulnerability to COVID-19 infection?

**DOI:** 10.2217/imt-2020-0077

**Published:** 2020-04-15

**Authors:** Joseph Kattan, Clarisse Kattan, Tarek Assi

**Affiliations:** 1 Hotel-Dieu de France University Hospital, Faculty of Medicine, Saint Joseph University, Beirut, Lebanon

**Keywords:** cancer, checkpoints inhibitors, coronavirus, COVID-19, immune therapy, immunosuppression

## Abstract

The severe acute respiratory syndrome coronavirus 2 (SARS-Cov-2) has been declared a pandemic by the WHO that claimed the lives of thousands of people within a few months. Cancer patients represent a vulnerable population due to the acquired immunodeficiency associated with anti-cancer therapy. Immune checkpoint inhibitors have largely impacted the prognosis of a multitude of malignancies with significant improvement in survival outcomes and a different, tolerable toxicity profile. In this paper, we assess the safety of ICI administration in cancer patients during the coronavirus pandemic in order to guide the usage of these highly efficacious agents.

Since it was first reported in the Wuhan region in China, the severe acute respiratory syndrome coronavirus 2 (SARS-Cov-2) has been declared a pandemic by the WHO with severe social and economic implications [1]. Until 25 March 2020, COVID-19 has affected more than 400,000 people around the globe and has claimed the lives of more than 18,000 persons [2]. While China, the origin site of the coronavirus spread, has succeeded in reducing the number of infected people, other countries have failed to halt the transmission of the virus with the struggle of their healthcare systems [3]. Generally, the clinical presentation of the COVID-19 infected patients is commonly encountered in cancer patients on a daily basis with a high incidence of fever, fatigue, dry cough and dyspnea while most common laboratory abnormalities include lymphopenia, prolonged prothrombin time and increased lactate dehydrogenase (LDH). So far, no vaccine or therapeutic strategy is yet available with the initial management of these patients involving optimal supportive care and oxygen supplementation for their respiratory failure symptoms [1].

Worldwide, cancer remains a heavy burden with more than 18 million cases diagnosed in 2018 according to the Globocan reports while the prevalence of cancer is largely beyond 43 million people [4]. Cancer patients might be more susceptible to a higher risk of infections and the COVID-19-related complications due to systemic immunodeficiency, mainly due to the effects of anticancer therapy [5–7]. Growing evidence supports the fact that avoiding immune destruction or immunoevasion is a new aspect of the hallmarks of cancer [8]. This conclusion is based on the theoretical idea that cells are constantly monitored by an active immune system that is responsible for the recognition and elimination of cancer cells and nascent tumors [8]. It is also thought that the chronic inflammation associated with cancer can help the development of an immunosuppressive tumor microenvironment, thus helping the tumor escape from immune surveillance [9]. However, not all cancer patients should be considered equally immunocompromised but particularly patients on active chemotherapy or those with hematological tumors such as leukemia and lymphomas.

## COVID-19 & cancer

In a report from China on 72,314 Covid-19-positive patients, the crude-fatality rate (CFR) was 2.3% among infected patients with higher mortality rates among those aged 70 years and older. Among cancer patients, the CFR was 5.6% [10]. On the other hand, the CFR in the Italian population was higher reaching 7.2%, which was attributed to the older age distribution and the different and selective testing strategy, mainly for symptomatic patients. In a small sample of Italian patients, 20.3% had an active cancer as a common comorbidity [11]. From another report from the Chinese population, 18 out of 1590 COVID-19 cases had history of active cancer. In these patients, there was a higher proportion of serious events occurrences (defined as the percentage of intensive care admissions with invasive ventilation or death) in comparison to those without cancer while older age represented the only risk factor for serious events [7]. Several strategies have been proposed by the authors to closely monitor cancer patients, including postponing chemotherapy or surgery in stable patients and a closer surveillance strategy in the elderly patients and those with multiple comorbidities [7]. However, these data should be cautiously interpreted due to relatively small sample size. Also, history of smoking should be considered as a predisposing factor in these patients with increased susceptibility to infection with COVID-19 since tobacco increases the expression of angiotensin-converting enzyme 2, a binding receptor for the SARS-Cov-2 [12]. Additionally, cancer is associated with an overexpression of immunosuppressive cytokines, reduced proinflammatory danger signals and enhanced functional immunosuppressive leukocyte population, which may induce a blunted immune system and increase the possibility of infectious complications [13].

## COVID-19 & immune checkpoint inhibitors

### Role of ICIs in cancer

On 17 March 2020, the National Health Service (NHS) published a report on the management of cancer patients during the coronavirus pandemic in which a large proportion of cancer patients were considered to be at utmost risk of infection by COVID-19. These patients were those receiving active chemotherapy or radiation therapy, those with hematological cancers or bone marrow transplants, as well as those receiving targeted therapy (protein kinase or PARP inhibitors) or immune therapy [14]. Interestingly, patients treated with immune checkpoint inhibitors (ICIs) were considered highly vulnerable if they were to be tested positive for the coronavirus infection. Since their introduction into the therapeutic arsenal against cancer, ICIs have revolutionized the treatment sequencing in the cancer management [15]. These monoclonal antibodies targeting immune checkpoints (PD1 and PD-L1), by their role in restoring the antitumor immunity through the reversal of immune escape or evasion, have led to a significant antitumor activity with confirmed impact on the duration of tumor response and prolonged overall survival. They have earned fast approvals across multiple cancer subtypes including melanoma, lung cancer, urological tumors, breast cancer and other solid tumors [16–20]. More importantly, these agents have earned several indications as monotherapy without association to chemotherapeutic agents, thus predisposing cancer patients to a different and more tolerable toxicity profile [16,17,19,21]. These agents have led to the occurrence of a new spectrum of immune-related adverse events (irAEs) ranging from moderate to severe and life-threatening ones. These side effects include a wide variety of adverse events such as endocrine abnormalities (thyroid dysfunction, adrenal insufficiency and hypophysitis), gastrointestinal events (colitis, hepatitis), and dermatological or respiratory events (interstitial pneumonitis), which merit a high index of suspicion with clear therapeutic strategies in order to prevent irreversible outcomes [22].

## Immunosuppression & ICIs

During the pandemic phase of the coronavirus there should be careful monitoring of cancer patients undergoing active therapy to prevent irreversible and life-threatening outcomes. In fact, cancer patients receiving cytotoxic chemotherapy are at high risk of developing infectious complications, mainly due to their impact on the myeloproliferative cells in the bone marrow but also on the rapidly dividing cells including the gut mucosa cells leading to the disruption of the protective barrier [23]. Therefore, patients receiving active chemotherapy will often develop neutropenia and up to 5–30% of patients might develop febrile neutropenia [23]. Also, patients undergoing hematopoietic stem cell transplantation or receiving chemotherapy for hematologic cancers are at the highest risk of developing prolonged neutropenia or febrile neutropenia episodes [23–25]. On the other hand, data on the hematological irAEs secondary to ICIs seems to be more comforting. Their occurrence is uncommon with different forms of adverse events including autoimmune thrombocytopenia and neutropenia, antibody-mediated hemolytic anemia and thrombotic thrombocytopenic purpura [22]. These adverse events highlight the crosstalk between the humoral and cellular immunity and the Treg-mediated self-tolerance [22]. In fact, ICIs might play a role in boosting pathogen-specific immune response in contrast to other immune checkpoints agonists such as Abatacept [26]. Their impact on the immune system may be beneficial in countering the immunosuppressive microenvironment and does not cause immunosuppression by itself [26]. Few cases have reported on severe ICI-related neutropenia, mostly secondary to anti-CTLA4 agent Ipilimumab but the overall rate of grade 3–5 rate of neutropenia remains low around 0.94% (overall occurrence around 0.3–1.07%) while the incidence of febrile neutropenia was 0.54% [27–31]. A small series by Finkel *et al.* analyzed the characteristics of 32 patients with immune-related neutropenia (irN). In these patients, the median time to onset of irN after the introduction of ICIs was 60 days with an incidence of febrile neutropenia of 50%. IR neutropenia resolved in 84% of cases after a combination of therapeutic agents including oral or intravenous steroids, granulocyte colony-stimulating factors and intravenous immunoglobulin [32]. That said, the incidence of severe neutropenia secondary to ICIs is very rare and can be controlled with an adapted therapeutic strategy if it occurs. Moreover, the instauration of prolonged immunosuppressive agents such as corticosteroids in a subset of patients with IrAEs might predispose to higher risk of infection and should also be closely monitored during the pandemic phase [33]. Few cases of infections secondary to the treatment of ICI-related side effects have been reported, since up to 10 and 50% of patients treated with ICI as monotherapy or combination therapy might develop irAEs [26,34]. There are also very few nonsolid data in the literature describing viral infections or reactivations as a complication to ICIs usage regardless of the development of irAEs such as varicella zoster infection, JC virus, hepatitis B, cytomegalovirus or Epstein–Barr virus [34,35].

## Conclusion

With the increasing risk of the COVID-19 pandemic, the management and selection of anti-cancer therapy in oncology patients including ICIs must be carefully balanced on a case-by-case scenario with the potential increase in the risk of complications or death from the coronavirus infection. Data on the immunosuppressive impact of ICIs in cancer patients are inconclusive but they seem to be more tolerable and reassuring than the heavy burden of hematological toxicities associated with other chemotherapeutic agents. Since we are not able to consider ICIs treatment as highly immunosuppressive, avoiding it in cancer patients to reduce coronavirus infections could deprive these patients from a highly active class of drugs. A special consideration should be given to patients treated for irAEs who are exposed to prolonged duration of immunosuppressive agents. While the world is preoccupied by the fight against coronavirus, adapted strategies and recommendations by the cancer community should be implemented to optimize the management of oncology patients. This could be achieved by a careful selection of the most efficacious anti-tumor weaponry with the lower risk of weaning the patients' immune system against a potential infection by the COVID-19. Real-world data from the COVID-19 affected areas are eagerly needed to assess the outcomes of oncology patients in order to enhance their therapeutic strategies.
